# Factors associated with the maintenance of breastfeeding for 6, 12, and 24 months in adolescent mothers

**DOI:** 10.1186/s12889-018-5585-4

**Published:** 2018-05-31

**Authors:** Mariana Muelbert, Elsa R. J. Giugliani

**Affiliations:** Post-Graduate Program in Child and Adolescent Health, Department of Pediatrics, Faculty of Medicine, Rua Ramiro Barcelos, 2400 2º andar, Porto Alegre, RS CEP: 90035003 Brazil

**Keywords:** Breastfeeding, Breastfeeding duration, Determinants of breastfeeding, Adolescent, Adolescent behavior, Cohort study

## Abstract

**Background:**

Previous studies have demonstrated that adolescent mothers present a higher risk of not breastfeeding or of early interruption of this practice. Considering the scarcity of studies investigating the determining factors of breastfeeding in adolescent mothers, and the absence of studies exploring the determining factors of breastfeeding maintenance for different periods of time in a single population of adolescent mothers, the aim of this research was to identify factors associated with breastfeeding maintenance for at least 6, 12, and 24 months in adolescent mothers.

**Methods:**

Data analysis from a randomised control trial involving adolescent mothers recruited at a university hospital in southern Brazil. Participants were followed through the first year of life of their infants and reassessed at 4–7 years. Factors associated with any breastfeeding for at least 6, 12, and 24 months were assessed using multivariate Poisson regression.

**Results:**

Data for 228, 237, and 207 mothers were available, respectively. Breastfeeding maintenance for at least 6, 12, and 24 months was observed in 68.4, 47.3, and 31.9% of the sample, respectively. Only one factor was associated with breastfeeding maintenance at all outcomes: infant not using a pacifier showed a higher probability of breastfeeding maintenance in the first 2 years. Maternal grandmother breastfeeding support and exclusive breastfeeding duration were associated with breastfeeding maintenance for 6 and 12 months. The other factors evaluated were associated with breastfeeding maintenance at only one of the time points assessed: 6 months, maternal skin color (black/brown); 12 months, female infant and partner breastfeeding support; and 24 months, older paternal age and multiparity**.**

**Conclusions:**

The present findings shed light upon barriers and facilitators of breastfeeding practices among adolescent mothers. In order to contribute to the challenge of increasing BF duration among adolescent mothers interventions aimed at boosting breastfeeding maintenance among this population should take into consideration the determining factors here identified. Additionally, breastfeeding education and support should be provided continuously as factors influencing these practices vary with time. Thus, support for adolescent mothers during the different stages of breastfeeding need to be tailored to have a positive impact on breastfeeding experience.

## Background

The positive impact of breastfeeding on child and maternal health, in both the short and long terms, and in both developing and developed countries, is widely recognized [1]. Nevertheless, breastfeeding rates in international and Brazilian settings are far from reaching optimal levels [1]. In Brazil, the last national survey on the prevalence of breastfeeding suggests that breastfeeding indicators did not advance in the last decade. The prevalence of exclusive breastfeeding in infants under 6 months and of any breastfeeding in infants under 24 months has remained unchanged, at about 37 and 52%, respectively. The only indicator showing improvement was the prevalence of breastfeeding in infants aged 21 to 23 months, which increased from 23.3% in 2006 to 31.8% in 2013 [2].

Some studies have pointed out that adolescent mothers show a lower prevalence of breastfeeding initiation and shorter breastfeeding duration [3–5] when compared with adult mothers; this finding has also been observed in Brazil [6–8]. Considering this scenario, the need to invest in breastfeeding promotion, protection, and support strategies targeted at younger mothers becomes evident. It is known that the impact of this type of intervention can vary greatly, depending on the characteristics of the intervention, including the setting where it is performed, the agents responsible for delivering it, the type of message conveyed, and number of exposures to the intervention, among other factors [9]. Moreover, in order for an intervention to be successful, it is necessary to take into consideration the determinants of early weaning and of breastfeeding maintenance in the target population, as these are known to vary and therefore may be different in adolescent mothers when compared with adult mothers [10]. Furthermore, the different factors associated with breastfeeding practices (breastfeeding initiation, exclusiveness and maintenance for given periods) should be carefully considered as well.

Considering the scarcity of studies investigating the determining factors of breastfeeding in adolescent mothers, and the absence of studies exploring the determining factors of breastfeeding maintenance for different periods of time in a single population of adolescent mothers, the objective of this study was to identify the factors associated with breastfeeding maintenance for at least 6, 12, and 24 months in a cohort of adolescent mothers.

## Methods

This study used data retrieved from the database of a randomized clinical trial. The original intervention aimed to increase the prevalence of exclusive breastfeeding and any breastfeeding and to improve complementary feeding practices among children of adolescent mothers; the intervention included the children’s maternal grandmothers, when cohabiting. It consisted of six counseling sessions on breastfeeding and healthy complementary feeding, the first one held at the maternity ward and the subsequent ones at the mothers’ homes. The mother-infant dyads were followed through the first year of life of their infants and reassessed when the children were 4–7 years old. Details on the methodology, intervention, and clinical trial results can be found elsewhere [11–14].

The study took place at the maternity ward of the Hospital de Clínicas de Porto Alegre between May 2006 and January 2008. Adolescent mothers and their respective infants were invited to participate. Maternal grandmothers could also participate whenever they cohabited with the mother-infant dyad. The hospital where the study was carried out is a Baby Friendly teaching hospital that assists primarily users of the Brazilian public health care system (approximately 3 thousand deliveries annually).

Mothers with the following characteristics were considered eligible for the study: age under 20 years, residence in the same municipality where the study took place), having given birth to healthy singleton newborn weighing 2500 g or more, and having initiated breastfeeding at the maternity ward. Mothers who could not stay in the rooming-in setting due to mother or newborn health problems were not included in the study. Also, because the intervention involved maternal grandmothers, adolescent mothers residing with their mothers-in-law (paternal grandmothers) were not included to avoid a confounding factor.

For the present study, sample size power was calculated a posteriori, considering the sample available for analysis. The following parameters were used: breastfeeding prevalence of 69.7% at 6 months, 50.1% at 12 months, and 32.2% at 24 months [12, 15], and a minimum relative risk of 1.25, 1.5, and 1.65, respectively, to assess associations between the variables and outcomes of interest. The sample available for assessing the outcome (breastfeeding) at 6, 12, and 24 months presented a power of at least 80% in the two-tailed hypothesis test, at a significance level of 5%.

Data collection occurred at different moments. Adolescent mothers and maternal grandmothers were interviewed separately at the maternity ward. This first interview focused on collecting sociodemographic data, information on prenatal care, delivery and previous experience with breastfeeding. Grandmothers answered a different questionnaire. The follow-up questionnaire was applied monthly up to the sixth month of life and every 2 months until they completed 1 year, either by telephone interview or home visit. This questionnaire included questions related to breastfeeding, complementary feeding, sources of breastfeeding support, pacifier use, and bottle use. To confirm the quality of the information collected, 5% of the participants were subjected to a second interview by the field researcher containing selected questions from the follow-up questionnaire.

When the children were between 4 and 7 years old, the participants were contacted once again by telephone, mail, or social networks; whenever necessary, the families were sought at home, at the latest address provided. Once located, participants were requested to visit the clinical research center at the hospital. At this occasion, data were collected on breastfeeding duration, feeding patterns, children’s weight and height, as well as updated data on the mother, child, and family.

For the scope of the present study, we aimed to access if the factors associated with breastfeeding maintenance for 24 month or more would be the same as the factors that influence breastfeeding maintenance for 12 and 6 months, thus we applied the same regression model for the different time points. Data available were submitted to a regression model in order to determine the factors associated with breastfeeding maintenance for at least 6 months, at least 12 months, and at least 24 months. World Health Organization definition for breastfeeding was used in the present study for the three outcomes assessed and therefore breastfeeding maintenance refers to children that received breastmilk (including expressed or donor milk), regardless of whether they were receiving other foods and liquids including non-human milk and formula [16].

**Fig. 1:**
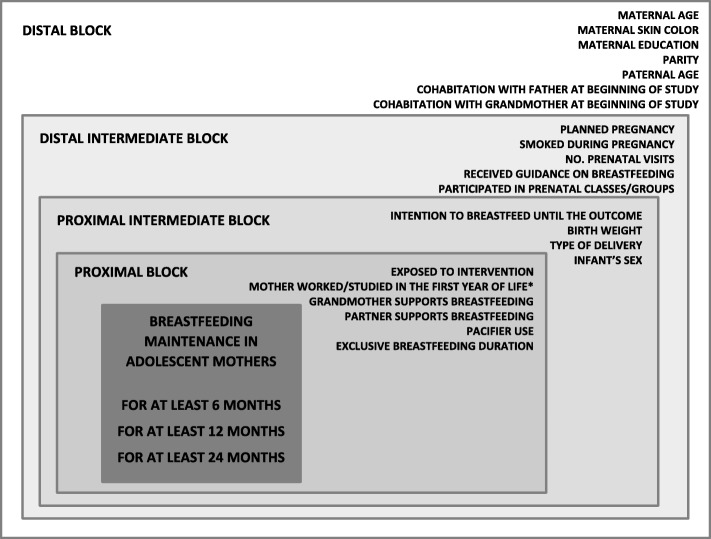
Hierarchical model used to identify factors associated with breastfeeding maintenance in adolescent mothers. * Variable included in the model at 12 and 24 months only

A regression model with a hierarchical approach was developed in which variables were distributed in blocks according to their relationship with the outcome [17]. The approach suggested by Boccolini et al. [18] was adopted, i.e., blocks were hierarchically organized based on the proximity of each exposure factor to the outcome. Therefore, different variables were distributed into four blocks. The first block (distal) comprised sociodemographic variables, maternal and family characteristics; the second block (distal intermediate) comprised variables related to the prenatal period; the third block (proximal intermediate), variables related to labor/delivery, the immediate postpartum period, and newborn characteristics, such as birth weight and sex; finally, the fourth block (proximal) included characteristics of the breastfeeding mothers and infants, including infant feeding patterns (Fig. 1). Because this study used data from a randomized clinical trial, the variable intervention was added to the proximal block of the model to rule out any eventual interference of the intervention on the results.

First, analyses were conducted to assess the possibility of multi-collinearity and to assess the association between the outcome and variables of interest in each block, using univariate Poisson regression. Variables in the first block (distal) that reached a level of significance of *p* < 0.20 in the univariate analysis were subjected to multivariate Poisson regression (intrablock analysis). Any variables reaching a significance level of *p* < 0.10 in the multivariate analysis remained in the model for adjustment of the next block. Subsequently, variables in the second block (distal intermediate) that reached *p* < 0.20 in the univariate analysis were subjected to multivariate Poisson regression along with the variables from the distal block that reached *p* < 0.10 in the previous multivariate analysis; and so on. This model predicted that, once a variable reached *p* < 0.10 in intrablock analysis, it would remain in the model until the end, adjusting associations between variables from the other blocks due to their possible role as confounding factors. The level of association between the different variables and the outcome was estimated using crude relative risk (RRc) and adjusted relative risk (RRa) and respective 95% confidence intervals (95% CI); associations were considered significant when *p* < 0.05. Missing data were managed as *listwise deletion* as regression model allows only entire records to enter the model for analysis. Statistical analyses were conducted using the Statistical Package for the Social Sciences version 21.0 (IBM Corp., 2012).

The present study was conducted according to the guidelines established by Resolution no. 466/2012 of the National Health Council, from the Brazilian Ministry of Health. Mothers and grandmothers received detailed information on the study and signed an informed consent form prior to any study procedures, and again before the last assessment. For adolescents under 18 years of age, consent was obtained from the adolescent mother and a parent/guardian. This research obtained approval from the Research Ethics Committee of Hospital de Clínicas de Porto Alegre (protocol no. 120249). The clinical trial was registered at ClinicalTrials.gov (NCT00910377).

## Results

A total of 323 adolescent mothers initiated the study, 257 (80%) participated in the 6th month follow-up, 237 (73%) remained up to 12 months, and 207 (64%) were available for the final evaluation (4–7 years). There was no statistically significant difference for maternal characteristics (skin color, age, educational level, number of prenatal visits, and number of children) and children’s characteristics (gender, birth weight, and mode of delivery) between those who completed the study and the participants who were lost to follow-up.

After exclusion of the cases lost to follow-up and participants with missing records, data from 228, 237, and 207 mothers were available for analysis of the factors associated with breastfeeding maintenance for at least 6, 12, and 24 months, respectively.

Maternal age at the beginning of the study ranged from 13 to 19 years, with a mean of 18 years; most mothers (52.9%) had at least 8 years of formal education, had a partner (84%) and cohabited with the infant’s father (62%); half of the adolescent mothers lived with the infant’s maternal grandmother. Mean paternal age was 22 years.

Breastfeeding maintenance for at least 6 months was observed in 68.4% of the sample, and for at least 12 and 24 months, in 47.3 and 31.9% of the mothers, respectively.

Table 1 shows the results of the multivariate analysis of associations between breastfeeding maintenance for at least 6 months and the variables selected, included in the model in blocks, as described above. Significant associations were observed between the outcome and maternal skin color (black/brown), maternal grandmother support of breastfeeding, never having used a pacifier, and longer exclusive breastfeeding duration (0.4% for each day of exclusive breastfeeding). Only one variable from the distal block and none from the two intermediate blocks were associated with the outcome.

**Table 1:** Factors associated with breastfeeding maintenance at 6 months in adolescent mothers. Analysis of variables subjected to Poisson hierarchical regression analysis

Distal block (*n* = 228)	N (%)	RRc (95%CI)	RRa (95%CI)
Maternal age			
≥18 years	124 (54.4)	1.17 (0.98–1.41)	1.15 (0.96–1.38)
Paternal age			
≥22 years	111 (48.6)	1.11 (0.95–1.33)	–
Maternal education			
≥8 years	123 (53.9)	0.92 (0.77–1.10)	–
Maternal skin color			
Black/brown	89 (39.0)	1.21 (1.02–1.43)^*^	1.22 (1.03–1.44)^*^
Parity			
Multiparous	33 (14.5)	0.97 (0.75–1.26)	–
Cohabitation with partner at beginning of study	135 (59.2)	1.13 (0.94–1.36)	1.07 (0.87–1.38)
Cohabitation with maternal grandmother at beginning of study	123 (53.9)	0.83 (0.70–0.99)^*^	0.87 (0.71–1.06)
Distal intermediate block			
Planned pregnancy	60 (26.3)	0.93 (0.76–1.15)	–
Smoked during pregnancy			
Did not smoke	150 (65.8)	1.14 (0.93–1.38)	–
Number of prenatal visits			
≥7 visits	144 (63.1)	1.18 (0.96–1.44)	1.17 (0.95–1.43)
Received prenatal guidance on breastfeeding	89 (39)	1.00 (0.84–1.20)	–
Participated in prenatal classes/groups	49 (21.5)	1.10 (0.90–1.33)	–
Proximal intermediate block			
Type of delivery			
Vaginal	179 (78.5)	0.98 (0.79–1.21)	-
Infant sex			
Female	112 (49.1)	1.09 (0.92–1.31)	-
Birth weight			
≥3200 g	110 (48.2)	0.99 (0.83–1.18)	-
Intention to breastfeed			
≥6 months	218 (95.6)	1.38 (0.74–2.59)	-
Proximal block			
Exposed to intervention	115 (50.4)	1.15 (0.96–1.37)	1.00 (0.86–1.18)
Maternal grandmother supports breastfeeding	163 (71.4)	1.44 (1.08–1.93)^*^	1.31 (1.04–1.64)^*^
Partner supports breastfeeding	164 (71.9)	1.48 (1.10–1.99)^*^	1.27 (0.99–1.63)
Pacifier use			
Does not use	102 (44.7)	1.73 (1.44–2.07)^**^	1.53 (1.30–1.80)^**^
Exclusive breastfeeding duration (days)	89 (39–180)^a^	1.005 (1.004–1.007)^**^	1.004 (1.002–1.005)^**^

^*^ *p* < 0.05; ^**^ *p* < 0.001

^a^Median (25th–75th interquartile range)

*RRc* crude relative risk, *RRa* adjusted relative risk, *95%CI* 95% confidence interval

In relation to the factors associated with breastfeeding maintenance for at least 12 months (Table 2), none of the variables from the distal and distal intermediate blocks showed significant associations with the outcome. Of the five variables showing significant associations, four were from the proximal block – maternal grandmother and partner support of breastfeeding, never having used a pacifier, and longer exclusive breastfeeding duration (0.2% for each day of exclusive breastfeeding) – and one was from the proximal intermediate block – infant female sex.

**Table 2:** Factors associated with breastfeeding maintenance at 12 months in adolescent mothers. Analysis of variables subjected to Poisson hierarchical regression analysis

Distal block (*n* = 237)	*N* (%)	RRc (95% CI)	RRa (95% CI)
Maternal age			
≥18 years	133 (56.1)	1.08 (0.82–1.42)	-
Paternal age			
≥22 years	117 (49.3)	1.05 (0.80–1.37)	-
Maternal education			
≥8 years	135 (56.9)	0.90 (0.69–1.18)	-
Maternal skin color			
Black/brown	84 (35.4)	1.22 (0.94–1.60)	-
Parity			
Multiparous	35 (14.7)	1.10 (0.77–1.57)	-
Cohabitation with partner at beginning of study	145 (61.2)	1.06 (0.8–1.40)	-
Cohabitation with maternal grandmother at beginning of study	132 (55.7)	0.89 (0.68–1.16)	-
Distal intermediate block			
Planned pregnancy	62 (26.1)	0.81 (0.58–1.14)	–
Smoked during pregnancy			
Did not smoke	163 (68.7)	1.30 (0.94–1.79)	–
Number of prenatal visits			
≥7 visits	153 (64.5)	1.00 (0.76–1.33)	-
Received prenatal guidance on breastfeeding	100 (42.2)	1.07 (0.81–1.40)	–
Participated in prenatal classes/groups	52 (21.9)	1.13 (0.83–1.53)	–
Proximal intermediate block			
Type of delivery			
Vaginal	186 (78.5)	0.86 (0.64–1.17)	-
Infant sex			
Female	117 (49.3)	1.31 (0.99–1.72)	1.32 (1.003–1.73)^*^
Birth weight			
≥3200 g	123 (51.9)	1.07 (0.82–1.40)	-
Intention to breastfeed			
≥12 months	190 (80.1)	1.29 (0.87–1.91)	1.31 (0.88–1.96)
Proximal block			
Exposed to intervention	111 (46.8)	0.73 (0.55–0.97)^*^	0.98 (0.78–1.23)
Mother worked/studied in the first year of life	34 (14.3)	1.30 (0.82–2.06)	–
Maternal grandmother supports breastfeeding	115 (48.5)	4.99 (2.98–8.37)^**^	2.14 (1.22–3.77)^*^
Partner supports breastfeeding	113 (47.6)	4.06 (2.56–6.45)^**^	1.63 (1.06–2.53)^*^
Pacifier use			
Does not use	98 (41.3)	3.15 (2.29–4.33)^**^	2.17 (1.55–3.03)^**^
Exclusive breastfeeding duration (days)	89 (39–189)^a^	1.007 (1.005–1.009)^**^	1.002 (1.00–1.004)^*^

^*^p < 0.05; ^**^ *p* < 0.001

^a^Median (25th–75th interquartile range)

*RRc* crude relative risk, *RRa* adjusted relative risk, *95%CI* 95% confidence interval

Finally, of the three variables associated with breastfeeding maintenance for at least 24 months (Table 3), two were from the distal block – paternal age ≥ 22 years and multiparity – and one was from the proximal block – never having used a pacifier.

**Table 3:** Factors associated with breastfeeding maintenance at 24 months in adolescent mothers. Analysis of variables subjected to Poisson hierarchical regression analysis

Distal block (*n* = 207)	N (%)	RRc (95%CI)	RRa (95%CI)
Maternal age			
≥18 years	116 (56.0)	1.21 (0.80–1.82)	-
Paternal age			
≥22 years	99 (47.8)	1.66 (1.10–2.51)^*^	1.59 (1.06–2.40)^*^
Maternal education			
≥8 years	110 (53.1)	1.36 (0. 90–2.05)	1.32 (0.88–1.99)
Maternal skin color			
Black/brown	78 (37.7)	1.22 (0.82–1.82)	-
Parity			
Multiparous	30 (14.5)	1.73 (1.13–2.66)^*^	1.79 (1.17–2.74)^*^
Cohabitation with partner at beginning of study	125 (60.4)	1.15 (0.76–1.74)	-
Cohabitation with maternal grandmother at beginning of study	109 (52.6)	0.95 (0.64–1.42)	-
Distal intermediate block			
Planned pregnancy	49 (23.6)	1.55 (0.88–2.72)	1.45 (0.84–2.51)
Smoked during pregnancy			
Did not smoke	140 (67.6)	1.50 (0.92–2.42)	1.47 (0.93–2.34)
Number of prenatal visits			
≥7 visits	134 (64.7)	0.99 (0.65–1.51)	-
Received prenatal guidance on breastfeeding	82 (39.6)	0.99 (0.66–1.49)	–
Participated in prenatal classes/groups	46 (22.2)	1.21 (0.78–1.89)	–
Proximal intermediate block			
Type of delivery			
Vaginal	154 (74.4)	1.07 (0.67–1.72)	-
Infant sex			
Female	106 (51.2)	1.22 (0.81–1.82)	–
Birth weight			
≥3200 g	98 (47.3)	0.82 (0.55–1.23)	–
Intention to breastfeed			
≥24 months	135 (65.2)	1.23 (0.79–1.90)	-
Proximal block			
Exposed to intervention	98 (47.3)	0.87 (0.58–1.30)	–
Mother worked/studied in the first year of life	48 (23.2)	1.36 (0.79–2.32)	–
Maternal grandmother supports breastfeeding	61 (29.4)	6.34 (3.17–12.68)^**^	3.33 (0.79–13.92)
Partner supports breastfeeding	60 (29.5)	6.52 (3.27–13.03)^**^	1.79 (0.49–6.52)
Pacifier use			
Does not use	98 (47.3)	4.13 (2.45–6.97) ^**^	2.05 (1.07–3.92)^*^
Exclusive breastfeeding duration (days)	59 (29–119)^a^	1.006 (1.003–1.010)^**^	1.0 (0.996–1.004)

^*^ *p* < 0.05;^**^ *p* < 0.001

^a^Median (25th–75th interquartile range)

*RRc* crude relative risk, *RRa* adjusted relative risk, *95%CI* 95% confidence interval

Table 4 presents the variables that showed association with at least one of the three outcomes assessed. Of the eight variables, five were associated with only one of the three outcomes, two with two outcomes – maternal grandmother support of breastfeeding and exclusive breastfeeding duration –, and only one variable was associated with all three outcomes – infant never having used a pacifier.

**Table 4:** Variables associated with breastfeeding maintenance in adolescent mothers

	Breastfeeding ≥6 months	Breastfeeding ≥12 months	Breastfeeding ≥24 months
Distal block			
Paternal age, ≥22 years			X
Maternal skin color, black/brown	X		
Multiparity			X
Proximal intermediate block			
Infant sex, female		X	
Proximal block			
Maternal grandmother supports breastfeeding	X	X	
Partner supports breastfeeding		X	
Pacifier use, never	X	X	X
Longer exclusive breastfeeding duration	X	X	

## Discussion

This is the first study to investigate factors associated with breastfeeding maintenance for 24 months in adolescent mothers and also for different periods of time in this population. The demonstration that the factors associated with breastfeeding maintenance may vary depending on the time frame assessed and that they show peculiarities in adolescent mothers adds new and relevant information to the existing state of the art.

We identified only two previous studies that also investigated factors associated with breastfeeding maintenance for 6, 12, and 24 months – one conducted in Croatia [19] and the other in the United States [20]. Both studies included women of all age groups and also observed changes in the factors associated with breastfeeding maintenance over time. However, of the determining factors assessed by Langellier et al. in the United States, four (intention to breastfeed prior to birth, breastfeeding at the maternity ward, being interviewed in Spanish, and mother not returning to work in the first 3 months) were positively associated with breastfeeding maintenance at the three time points assessed, compared to only one factor in the Croatian study by Zakarija-Grkovic et al. (antenatal course attendance). In our study, not using a pacifier was the only factor associated with breastfeeding maintenance at the three time points assessed.

The association between pacifier use and duration of both exclusive breastfeeding and any breastfeeding has been widely investigated, with evidence suggesting that pacifier use can have negative effects on breastfeeding duration [21, 22]. In contrast, recent research with American mothers has demonstrated that among young mothers, aged 18–19 years, the use of pacifier was positively associated with breastfeeding practice at 1 week but this association was inverse among older mothers aged 30 or more [23]. However, no effect on breastfeeding maintenance was assessed limiting comparisons with the present study. Thus, the mechanisms underlying this association are not fully understood and could be affected by maternal age. In this sense, our study was the first to demonstrate a negative association between pacifier use and breastfeeding maintenance in adolescent mothers in Brazil: infant not using a pacifier in our population increased the chance of maintaining breastfeeding for at least 6 months by 1.5 and doubled the chance of breastfeeding maintenance for at least 12 or 24 months.

It is worth to mention that even though pacifier use might be indicated to reduce incidence of Sudden Unexpected Deaths in Infancy (SUDI) and Sudden Infant Death Syndrome (SIDS) when given at naptime or bedtime in some countries, the American Academy of Pediatrics and other policy makers in Canada, United Kingdom, New Zealand and Australia recognize that the introduction of pacifiers should be delayed until 4 to 6 weeks to avoid interference with establishment of breastfeeding. Breastfeeding is associated with reduced risk of SUDI and SIDS, however the mechanism behind the protective effects of pacifiers on prevention of SUDI and SIDS remain unclear and therefore the indication for the use of pacifier merits caution to prevent a negative impact on the establishment of breastfeeding [24–28].

Maternal grandmother support of breastfeeding showed a significant association with breastfeeding maintenance for at least 6 and 12 months, but not for 24 months, despite the magnitude of the association. This finding is not surprising, as receiving breastfeeding support from different sources – especially from the adolescent mother’s mother – is known to foster the practice [29, 30]. A recent systematic review demonstrated that the opinion of the infant’s maternal grandmother regarding breastfeeding influences maternal decisions: when positive, it may increase by 12% the likelihood of the mother initiating breastfeeding; when negative, it may reduce by 70% the likelihood of breastfeeding [31].

Partner breastfeeding support was also positively associated with breastfeeding maintenance for at least 12 months, but not for 6 or 24 months. Even though there is consensus in the literature on the importance for breastfeeding of the support provided by the infant’s father, some studies have suggested that fathers are not always prepared to provide this support. For instance, a Brazilian study involving adolescent mothers found a 1.6 higher risk of breastfeeding interruption before 6 months when the adolescent mother was married [10]; another study, also conducted in Brazil, identified cohabitation with partner as a risk factor for breastfeeding maintenance for at least 24 months in women from all age groups [32]. Moreover, experiencing partner violence was an important risk factor for breastfeeding interruption before 6 months in a study carried out with adolescent mothers in the United States [33].

Another partner characteristic that showed a positive association with breastfeeding maintenance in our study was paternal age, however only at 24 months. To the authors’ knowledge, this was the first study investigating factors associated with breastfeeding using multivariate analysis that identified an association between paternal age and breastfeeding duration. Even though maternal age is among the factors most widely studied, paternal age tends to be neglected. More studies are needed that investigate this association in women of all age groups, to clarify the factors involved. It is possible that younger paternal age is related with lower paternal interest in caring for the infant, and also with poor knowledge of the positive effects of prolonged breastfeeding on both infant and maternal health, especially among teenage couples. Finally, it is also likely that younger fathers see breastfeeding as a barrier to resuming full sexual activity after the infant’s birth, a fact that may be interpreted differently by older fathers. It is interesting to observe that, among the few studies that have explored paternal age as a possible risk factor for breastfeeding, all failed to identify a significant association [34–36]; however, none of them had adolescent mothers as the target population. This finding underscores the relevance of including the infant’s father or mother’s partner in breastfeeding promotion strategies.

In addition to paternal age, another factor that showed association with breastfeeding maintenance at 24 months only was the mother having an older child. There have been reports of positive associations between multiparity and exclusive breastfeeding in adolescents [7], but none of the two studies that assessed this variable in adolescent mothers confirmed the association between any breastfeeding duration and multiparity [10, 33]. The experience acquired with older children may help the mother overcome any difficulties during pregnancy, delivery, puerperium, and also during breastfeeding [37, 38]. Studies indicate that mothers who had a positive experience with breastfeeding are more likely to breastfeed their subsequent child [39–42], suggesting that effort should be made to support first-time mothers’ with breastfeeding as it might have positive impact on subsequent pregnancies and breastfeeding practices. Furthermore, women who have experienced motherhood before may have an increased knowledge of infant care, introducing complementary feeding at a more appropriate time and maintaining breastfeeding for longer [43].

In addition to maternal grandmother support of breastfeeding, another factor positively associated with breastfeeding maintenance for two of the three time periods assessed, namely 6 and 12 months, was exclusive breastfeeding duration. This association has been described previously in American adolescents: having exclusively breastfed reduced by almost 40% the risk of interrupting breastfeeding before 6 months in that population [33]. A similar finding was observed in another study, comprising women of all ages: the introduction of formula during the infant’s first month of life doubled the risk of interrupting breastfeeding before 12 months [44]. The association between introduction of complementary feeding to the breastfed infant and shorter breastfeeding duration is biologically plausible: first, there is a decrease in milk supply as the infant starts to breastfeed less often – a natural phenomenon when they start to receive other foods [45]; second, nipple confusion may occur, as bottles are usually the primary vehicle for the intake of water, tea, juices, and other milks at this age [46]. Furthermore, it is possible that the women who are committed to exclusively breastfeed their babies for longer are also the ones who will more rigorously observe breastfeeding duration recommendations.

Finally, two other factors contributed to breastfeeding maintenance in adolescent mothers: maternal skin color (black/brown) and female infant sex. These characteristics have been previously identified as protective factors for breastfeeding in the Brazilian population [15, 47, 48] where black and brown skin colors are a reflection of ethnicity. Nevertheless, this is the first time these findings are described in adolescent mothers that choose to breastfeed, as the two previous studies involving adolescent mothers failed to find associations between breastfeeding and skin color/race [33] or infant sex [10]. It is interesting to observe that these two factors are closely related to social and cultural disparities, and therefore vary greatly across populations. In some countries, black women breastfeed for shorter periods than their white counterparts – e.g., in the United States [49]; in others, male infants tend to be privileged with regard to breastfeeding – e.g., in Timor-Leste [50].

Our study included adolescent mothers only, and therefore it is not possible to compare the factors associated with breastfeeding duration in our population with the findings reported for adult mothers at the same time frames. However, looking at the results of a study conducted by the same group of authors with women of all ages, selected at the same hospital, and employing similar methodology could be useful to compare factors associated with breastfeeding maintenance for at least 24 months. In that study, five variables were positively associated with the outcome: not cohabiting with the infant’s father, mother staying at home in the first semester after birth, not using a pacifier, postponed introduction of water, tea, and complementary feeding [32]. Therefore, the only factor common to both studies was not using a pacifier. Parity was associated with breastfeeding maintenance for at least 24 months among adolescent mothers only, and paternal age was not explored in that study.

Some limitations of this study should be addressed. For example, the fact that this analysis relied on data obtained from a randomized clinical trial can be considered as a possible disadvantage. To minimize this limitation, we added the variable intervention to the proximal block of the regression model, in order to ensure that the associations observed were independent of group allocation in the original trial. Moreover, because the information on breastfeeding for at least 24 months was collected 4 to 7 years after birth, there is a possibility of memory bias. Nonetheless, we believe that this type of bias is unlikely as breastfeeding duration was expressed using two distinct time frames (< 24 months or ≥ 24 months), and the time elapsed after the outcome varied from 2 to 5 years, at the most. Another limitation of the study is the attrition rate faced during the follow-up phase common to studies that require tracking down participants for follow-up assessments. Even though active search for finding participants did not lead to fewer losses, this limitation did not affect the present findings as the characteristics of participants did not differed between those who completed the study and those lost during follow-up.

## Conclusion

The factors that influence breastfeeding duration modify as lactation evolves. Adolescent mothers face many challenges during their motherhood experience that appear to change as the child grows older and she returns to social and work activities. We believe that the results of this study can contribute to the challenge of increasing breastfeeding duration by individual-centered approach [51], i.e., by taking into consideration the associated factors here identified during planning and implementation of strategies targeted at adolescent mothers. In such strategies, it would be important to address the following topics: information on pacifier use, providing breastfeeding education before birth to both expecting mothers and family members; the importance of breastfeeding support from the mother’s partner and close family members, especially the infant’s grandmothers, including these subjects in the interventions whenever possible (especially younger fathers); and the importance of exclusive breastfeeding in the first 6 months of life of the infant. Additionally, we recommend that breastfeeding education and support should be provided continuously, prenatally and beyond, as we have identified that the factors influencing this practices changes with time and with the expected duration of breastfeeding practices. It is also necessary to bear in mind that our study has identified that white and primiparous mothers tend to breastfeed for shorter periods of time and more attention should be given to them when designing strategies to improve breastfeeding practices. Moreover, we have identified that the factors associated with breastfeeding maintenance change over time, which means giving more emphasis on one or another factor, depending on the stage of breastfeeding being assessed. All in all, we expect that these findings will contribute to improve the knowledge of aspects still little explored of the determinants of breastfeeding among younger mothers.
